# The chromosomal genome sequence of the mauve stinger jellyfish, *Pelagia noctiluca* (Forsskål, 1775) (Semaeostomeae: Pelagiidae)

**DOI:** 10.12688/wellcomeopenres.27407.1

**Published:** 2026-08-27

**Authors:** Lucas Leclère, Manon Boosten, Michael N Dawson, Graeme Oatley, Elizabeth Sinclair, Eerik Aunin, Noah Gettle, Camilla Santos, Michael Paulini, Haoyu Niu, Victoria McKenna, Rebecca O’Brien, Wellcome Sanger Institute Tree of Life Management, Samples and Laboratory team, Wellcome Sanger Institute Scientific Operations: Sequencing Operations, Wellcome Sanger Institute Tree of Life Core Informatics team, EBI Aquatic Symbiosis Genomics Data Portal Team, Aquatic Symbiosis Genomics Project Leadership

**Affiliations:** 1 Sorbonne Université, CNRS, Biologie Intégrative des Organismes Marins (BIOM), Banyuls-sur-Mer, France; 2 Sorbonne Université, CNRS, Laboratoire de Biologie du Développement de Villefranche-sur-Mer (LBDV), Villefranche-sur-Mer, France; 3 University of California Merced Dawson Lab, Merced, California, USA; 4 Tree of Life Programme, Wellcome Sanger Institute, Hinxton, England, UK

**Keywords:** Pelagia noctiluca, Mauve Stinger, genome sequence, chromosomal, Semaeostomeae

## Abstract

We present a genome assembly from a specimen of *Pelagia noctiluca* (Mauve Stinger; Cnidaria; Scyphozoa; Semaeostomeae; Pelagiidae). The genome sequence has a total length of 357.08 megabases. Most of the assembly (94.91%) is scaffolded into 21 chromosomal pseudomolecules. The mitochondrial genome has also been assembled, with a length of 16.7 kilobases. Gene annotation of this assembly by Ensembl identified 19 573 protein-coding genes.

## Species taxonomy

Eukaryota; Opisthokonta; Metazoa; Eumetazoa; Cnidaria; Scyphozoa; Semaeostomeae; Pelagiidae; *Pelagia*; *Pelagia noctiluca* (Fabricius, 1775) (NCBI:txid400838).

## Background

The medusa *Pelagia noctiluca* (Forsskål, 1775), also called the mauve stinger, is one of the most studied scyphozoan species due to its global distribution ( OBIS, 2024), unique life history, and tendency to form problematic outbreaks that disrupt commercial and recreational activities in coastal waters ( Marambio *et al.*, 2021). *Pelagia noctiluca* has a holoplanktonic life history, a rare feature among epipelagic scyphozoan medusae, allowing it to inhabit both coastal and oceanic regions ( Boosten *et al.*, 2025). Its range extends from the warm tropical waters, such as in the Gulf of Mexico ( Graham *et al.*, 2003) to colder regions, such as the North Sea ( Licandro *et al.*, 2010; Russell, 1970), or the South Atlantic Ocean ( Miller *et al.*, 2012). It is known as the most abundant scyphozoan medusa in the Mediterranean Sea ( Canepa *et al.*, 2014).

*Pelagia noctiluca* lacks a polyp stage. Once fertilised, the eggs develop directly into ephyrae ( Helm *et al.*, 2015), which then form juvenile medusae ( Ballesteros *et al.*, 2021). The umbrella of mature medusae, which can measure more than 20 cm in diameter, features medium-sized warts on the outer side, eight tentacles and eight rhopalia emerging alternately from clefts between lappets at the bell margin, and a circular coronal muscle on the inner side ( Ballesteros *et al.*, 2021; Russell, 1970). In laboratory conditions, the full life cycle can be completed in about three months ( Ballesteros *et al.*, 2022; Ramondenc *et al.*, 2019). As its name suggests (noctiluca – ‘shines by night’), this species is known for its bioluminescence ( Russell, 1970), generated and propagated throughout the body as a response to external stimuli ( Herring, 1990). Karyotypic analyses indicate that *Pelagia noctiluca* is a diploid organism with a haploid number of *n* = 21 and that there is an XY sex-determining mechanism ( Vitturi *et al.*, 1994).

This species was selected as a non-photosymbiotic reference taxon for the “Symbioses in 3D: Diversity and Dynamics in Pelagic Symbioses across the Tree of Life” project. Nonetheless, its unique position as an epipelagic holoplanktonic scyphozoan also makes its microbiome and cobionts of direct interest ( Tinta *et al.*, 2012). As a globally distributed taxon with some genetic patchiness ( Ale *et al.*, 2019; Glynn *et al.*, 2016; Miller *et al.*, 2012; Stopar *et al.*, 2010), there is potential for genotype-by-environment interactions in shaping microbiomes.

**Figure 1.**
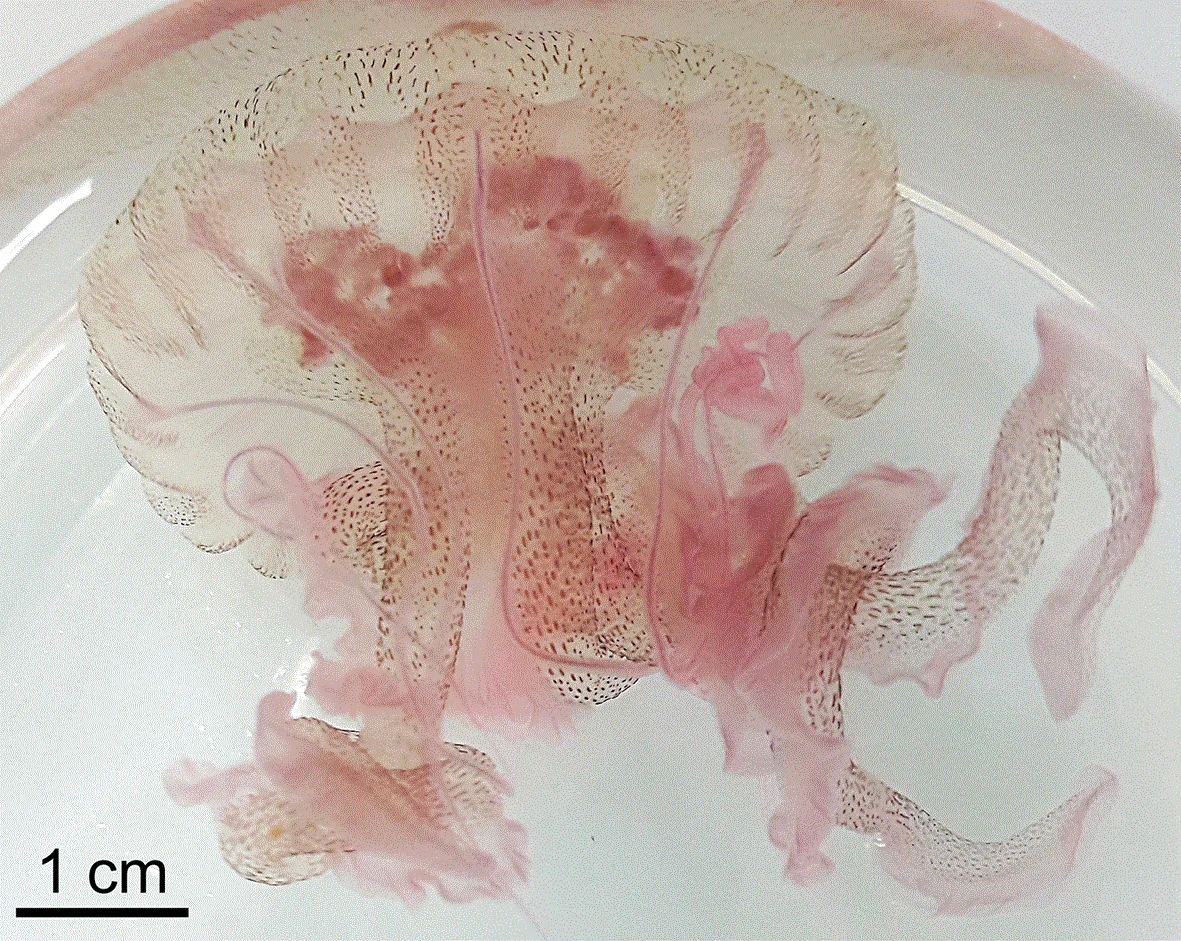
Photograph of the *Pelagia noctiluca* specimen (jsPelNoct2) used for genome sequencing.

This chromosome-level genome generated from a *Pelagia noctiluca* adult female ( Figure 1) collected in Villefranche-sur-Mer (France) will be broadly used for population genomics studies and for uncovering the biology of this significant species. Its peculiar direct development will be used to better understand life cycle evolution in cnidarians. Tools are currently being developed to establish this species as a relevant developmental genomics model, promising to reveal many aspects of scyphozoan biology.

## Methods

### Sample acquisition

A mature female medusa of *Pelagia noctiluca* (specimen ID PORT0000303, ToLID jsPelNoct2; Figure 1) was collected at the sea surface with a hand net in the bay of Villefranche-sur-Mer, France (latitude 43.673, longitude 7.3282) between 7 and 9 AM on 2022-09-05. The specimen was identified by LL and MB and starved for 24 hours in the laboratory in 20 L buckets of Millipore-filtered natural seawater at 18 °C. The oral arms, tentacles, and gonads were dissected in a Petri dish into 12 samples (four samples containing pieces of oral arms, two samples containing four tentacles each, and two samples containing pieces of gonads). These samples were then immediately transferred into cryotubes, snap-frozen in liquid nitrogen and transferred to a −80 °C freezer.

### Nucleic acid extraction

Protocols for high molecular weight (HMW) DNA extraction developed at the Wellcome Sanger Institute (WSI) Tree of Life Core Laboratory are available on protocols.io ( Howard *et al.*, 2025). The jsPelNoct2 sample was weighed and triaged to determine the appropriate extraction protocol. Tissue from the oral arm was homogenised by powermashing using a PowerMasher II tissue disruptor. HMW DNA was extracted using the Manual Modified Omega Bio-Tek protocol. DNA was sheared into an average fragment size of 12–20 kb following the Megaruptor®3 for LI PacBio protocol. Sheared DNA was purified by automated SPRI (solid-phase reversible immobilisation). The concentration of the sheared and purified DNA was assessed using a Nanodrop spectrophotometer and Qubit Fluorometer using the Qubit dsDNA High Sensitivity Assay kit. Fragment size distribution was evaluated by running the sample on the FemtoPulse system. For this sample, the final post-shearing DNA had a Qubit concentration of 21.0 ng/μL, with a fragment size of 14.7 kb. The 260/280 spectrophotometric ratio was 2.01, and the 260/230 ratio was 2.88.

RNA was extracted from oral arm tissue of the jsPelNoct2 specimen in the Tree of Life Laboratory at the WSI using the RNA Extraction: Automated MagMax™ *mir*Vana protocol. The RNA concentration was assessed using a Nanodrop spectrophotometer and a Qubit Fluorometer using the Qubit RNA Broad-Range Assay kit. Analysis of the integrity of the RNA was done using the Agilent RNA 6000 Pico Kit and Eukaryotic Total RNA assay.

### PacBio HiFi library preparation and sequencing

Library preparation and sequencing were performed at the WSI Scientific Operations core. Libraries were prepared using the SMRTbell Prep Kit 3.0 (Pacific Biosciences, California, USA), following the manufacturer’s instructions. The kit includes reagents for end repair/A-tailing, adapter ligation, post-ligation SMRTbell bead clean-up, and nuclease treatment. Size selection and clean-up were performed using diluted AMPure PB beads (Pacific Biosciences). DNA concentration was quantified using a Qubit Fluorometer v4.0 (ThermoFisher Scientific) and the Qubit 1X dsDNA HS assay kit. Final library fragment size was assessed with the Agilent Femto Pulse Automated Pulsed Field CE Instrument (Agilent Technologies) using the gDNA 55 kb BAC analysis kit.

The sample was sequenced on a Revio instrument (Pacific Biosciences, California, USA). Prepared libraries selected for multiplexing were pooled based on genome size, library molarity, and the intended plex level. Primers were annealed and polymerases were bound to generate circularised complexes, following the manufacturer’s instructions. Complexes were purified using SMRTbell beads, diluted to the Revio loading concentration, and spiked with a Revio sequencing internal control. The pooled libraries were sequenced on a Revio 25M Plex SMRT cell in a 4-plex run. SMRT Link software (Pacific Biosciences), a web-based workflow manager, was used to configure and monitor the run and to carry out primary and secondary data analysis.

### Hi-C

***Sample preparation and crosslinking***

The Hi-C sample was prepared from 20–50 mg of frozen oral arm tissue of the jsPelNoct2 sample using the Arima-HiC v2 kit (Arima Genomics). Following the manufacturer’s instructions, tissue was fixed and DNA crosslinked using TC buffer to a final formaldehyde concentration of 2%. The tissue was homogenised using the Diagnocine Power Masher-II. Crosslinked DNA was digested with a restriction enzyme master mix, biotinylated, and ligated. Clean-up was performed with SPRISelect beads before library preparation. DNA concentration was measured with the Qubit Fluorometer (Thermo Fisher Scientific) and Qubit HS Assay Kit. The biotinylation percentage was estimated using the Arima-HiC v2 QC beads.

***Hi-C library preparation and sequencing***

Biotinylated DNA constructs were fragmented using a Covaris E220 sonicator and size selected to 400–600 bp using SPRISelect beads. DNA was enriched with Arima-HiC v2 kit Enrichment beads. End repair, A-tailing, and adapter ligation were carried out with the NEBNext Ultra II DNA Library Prep Kit (New England Biolabs), following a modified protocol where library preparation occurs while DNA remains bound to the Enrichment beads. Library amplification was performed using KAPA HiFi HotStart mix and a custom Unique Dual Index (UDI) barcode set (Integrated DNA Technologies). Depending on sample concentration and biotinylation percentage determined at the crosslinking stage, libraries were amplified with 10–16 PCR cycles. Post-PCR clean-up was performed with SPRISelect beads. Libraries were quantified using the AccuClear Ultra High Sensitivity dsDNA Standards Assay Kit (Biotium) and a FLUOstar Omega plate reader (BMG Labtech).

Prior to sequencing, libraries were normalised to 10 ng/μL. Normalised libraries were quantified again to create equimolar and/or weighted 2.8 nM pools. Pool concentrations were checked using the Agilent 4200 TapeStation (Agilent) with High Sensitivity D500 reagents before sequencing. Sequencing was performed using paired-end 150 bp reads on the Illumina NovaSeq 6000.

### RNA library preparation and sequencing

Libraries were prepared using the NEBNext® Ultra II Directional RNA Library Prep Kit for Illumina (New England Biolabs), following the manufacturer’s instructions. Poly(A) mRNA was isolated from 100 ng of total RNA using oligo (dT) beads, converted to cDNA, and uniquely indexed; 14 PCR cycles were performed. Libraries were prepared with an intended fragment size of 100–300 bp. Libraries were quantified, normalised, and pooled equimolarly to a final concentration of 2.8 nM before dilution to 150 pM for loading. Sequencing was carried out on the Illumina NovaSeq X, generating paired-end reads.

### Genome assembly

Prior to assembly of the PacBio HiFi reads, a database of *k*-mer counts ( *k* = 31) was generated from the filtered reads using FastK. GenomeScope2 ( Ranallo-Benavidez *et al.*, 2020) was used to analyse the *k*-mer frequency distributions, providing estimates of genome size, heterozygosity, and repeat content.

The HiFi reads were assembled using Hifiasm ( Cheng *et al.*, 2021) with the --primary option. Haplotypic duplications were identified and removed using purge_dups ( Guan *et al.*, 2020). The Hi-C reads ( Rao *et al.*, 2014) were mapped to the primary contigs using bwa-mem2 ( Vasimuddin *et al.*, 2019), and the contigs were scaffolded in YaHS ( Zhou *et al.*, 2023) with the --break option for handling potential misassemblies. The scaffolded assemblies were evaluated using Gfastats ( Formenti *et al.*, 2022), BUSCO ( Manni *et al.*, 2021) and MerquryFK ( Rhie *et al.*, 2020).

The mitochondrial genome was assembled using MitoHiFi ( Uliano-Silva *et al.*, 2023). The MitoHiFi reference was the mitochondrial genome of *Pelagia noctiluca* (NC_080358.1).

### Assembly curation

The assembly was decontaminated using the Assembly Screen for Cobionts and Contaminants ( ASCC) pipeline. TreeVal was used to generate the flat files and maps for use in curation. Manual curation was conducted primarily in PretextView and HiGlass ( Kerpedjiev *et al.*, 2018). Scaffolds were visually inspected and corrected as described by Howe *et al*. (2021). Manual corrections included 28 breaks, 43 joins, and removal of 69 haplotypic duplications. This reduced the scaffold count by 22.6%, increased the scaffold N50 by 31.5%, and reduced the total assembly length by 10.1%. The curation process is described at sanger-tol/curation-resources . PretextSnapshot was used to generate a Hi-C contact map of the final assembly.

### Assembly quality assessment

The Merqury.FK tool ( Rhie *et al.*, 2020) was run in a Singularity container ( Kurtzer *et al.*, 2017) to evaluate *k*-mer completeness and assembly quality for the primary assembly, the alternate haplotig set, and their union using the *k*-mer database ( *k* = 31) computed prior to genome assembly. The analysis outputs included assembly QV scores and completeness statistics.

The genome was analysed using the BlobToolKit pipeline, a Nextflow implementation of the earlier Snakemake version ( Challis *et al.*, 2020). PacBio reads were aligned to the assembly using minimap2 ( Li, 2018) and processed with SAMtools ( Danecek *et al.*, 2021) to generate coverage tracks. The pipeline runs BUSCO ( Manni *et al.*, 2021) using lineages identified from the NCBI Taxonomy ( Schoch *et al.*, 2020). For the three basal BUSCO lineages, eukaryota, bacteria and archaea, BUSCO genes are aligned to the UniProt Reference Proteomes database ( Bateman *et al.*, 2023) using DIAMOND BLASTp ( Buchfink *et al.*, 2021). The genome assembly is divided into chunks based on the density of BUSCO genes from the closest taxonomic lineage, and each chunk is aligned to the UniProt Reference Proteomes database using DIAMOND BLASTx. Sequences without DIAMOND BLASTx hits are extracted using seqtk and aligned to the NT database using BLASTn ( Altschul *et al.*, 1990). The outputs are consolidated into a BlobDir for visualisation in BlobToolKit. The BlobToolKit pipeline was developed using nf-core tooling ( Ewels *et al.*, 2020) and MultiQC ( Ewels *et al.*, 2016), with containerisation through Docker ( Merkel, 2014) and Singularity ( Kurtzer *et al.*, 2017).

## Genome sequence report

### Sequence data

**Figure 2.**
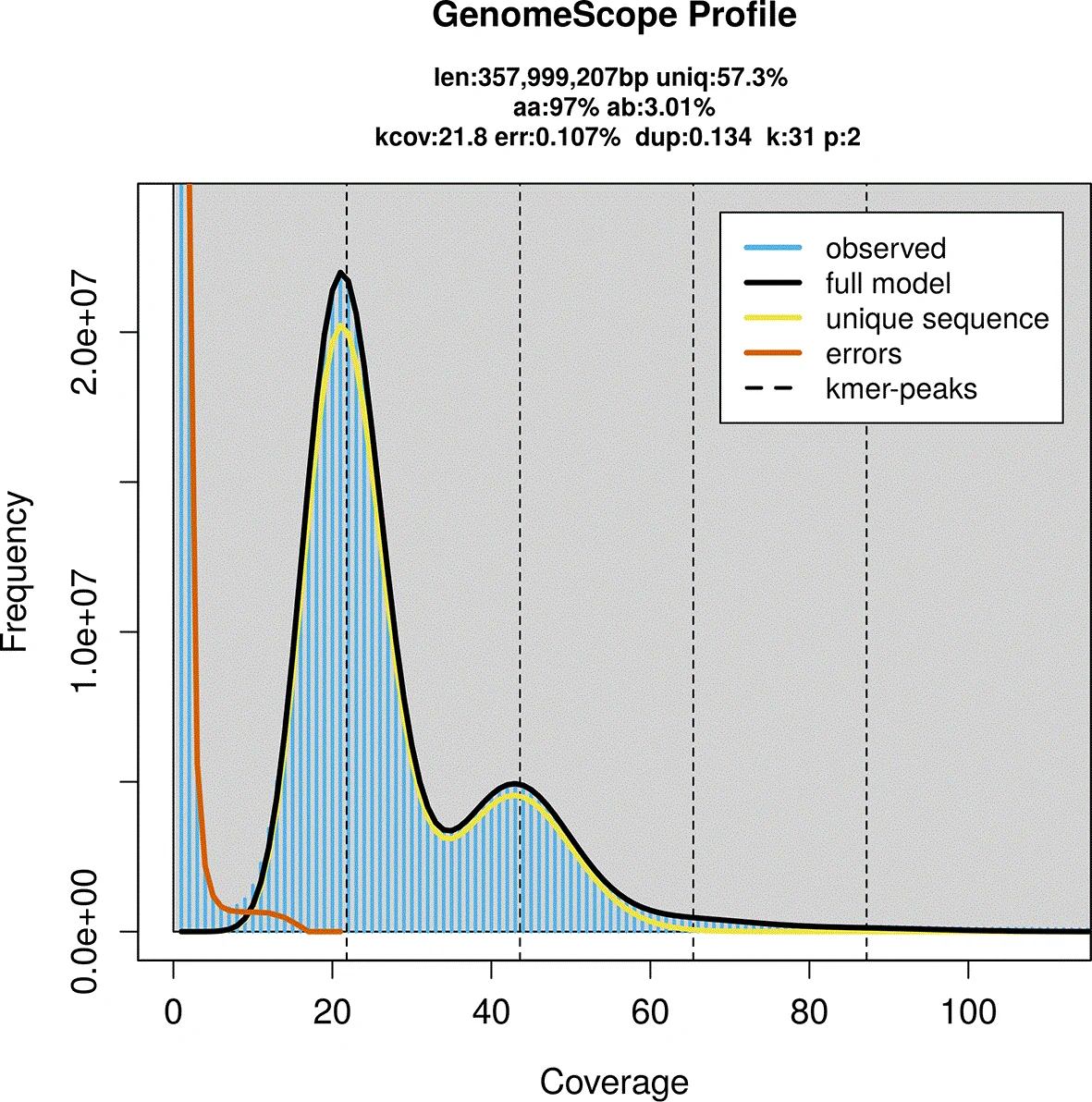
Frequency distribution of *k*-mers generated using GenomeScope2. The plot shows observed and modelled *k*-mer spectra, providing estimates of genome size, heterozygosity, and repeat content based on unassembled sequencing reads.

PacBio sequencing of the *Pelagia noctiluca* specimen generated 16.18 Gb (gigabases) from 1.57 million reads, which were used to assemble the genome. GenomeScope2.0 analysis using a *p* = 2 model gave a *k*-mer-based genome size estimate of 358.68 Mb, with a heterozygosity of 3.01% and repeat content of 42.70% ( Figure 2). These estimates guided expectations for the assembly. Based on the estimated genome size, the sequencing data provided approximately 44× coverage. Hi-C sequencing produced 116.15 Gb from 384.59 million reads, which were used to scaffold the assembly. RNA sequencing data were also generated and are available in public sequence repositories. Table 1 summarises the specimen and sequencing details.

**Table 1.** Specimen and sequencing data for *Pelagia noctiluca* (BioProject PRJEB71881).

Platform	PacBio HiFi	Hi-C	RNA-seq
**ToLID**	jsPelNoct2	jsPelNoct2	jsPelNoct2
**Specimen ID**	PORT0000303	PORT0000303	PORT0000303
**BioSample (source individual)**	SAMEA111439783	SAMEA111439783	SAMEA111439783
**BioSample (tissue)**	SAMEA111439802	SAMEA111439802	SAMEA111439797
**Tissue**	oral arm	oral arm	oral arm
**Instrument**	Revio	Illumina NovaSeq 6000	Illumina NovaSeq X
**Run accessions**	ERR12510611	ERR12507428	ERR13669962
**Read count total**	1.57 million reads	384.59 million read pairs	47.96 million read pairs
**Base count total**	16.18 Gb	116.15 Gb	14.48 Gb

### Assembly statistics

The assembly strategy prioritised a complete and contiguous primary pseudohaplotype. Contigs corresponding to residual alternate haplotigs were also deposited in INSDC databases. The final primary assembly has a total length of 357.08 Mb in 431 scaffolds, with 84 gaps, and a scaffold N50 of 16.77 Mb ( Table 2). The alternate haplotig set has a total length of 430.96 Mb in 1 968 contigs, with a contig N50 of 1.37 Mb.

**Table 2.** Genome assembly data for *Pelagia noctiluca.*

Genome assembly	Primary assembly
**Assembly name**	jsPelNoct2.1
**Assembly accession**	GCA_963924455.1
**Alternate haplotype accession**	GCA_963924465.1
**Assembly level**	chromosome
**Span (Mb)**	357.08
**Number of chromosomes**	21
**Number of contigs**	515
**Contig N50**	6.03 Mb
**Number of scaffolds**	431
**Scaffold N50**	16.77 Mb
**Organelles**	Mitochondrial genome: 16.70 kb

The scaffold count follows the NCBI assembly statistics and excludes organelle assemblies, which are reported separately where present.

**Figure 3.**
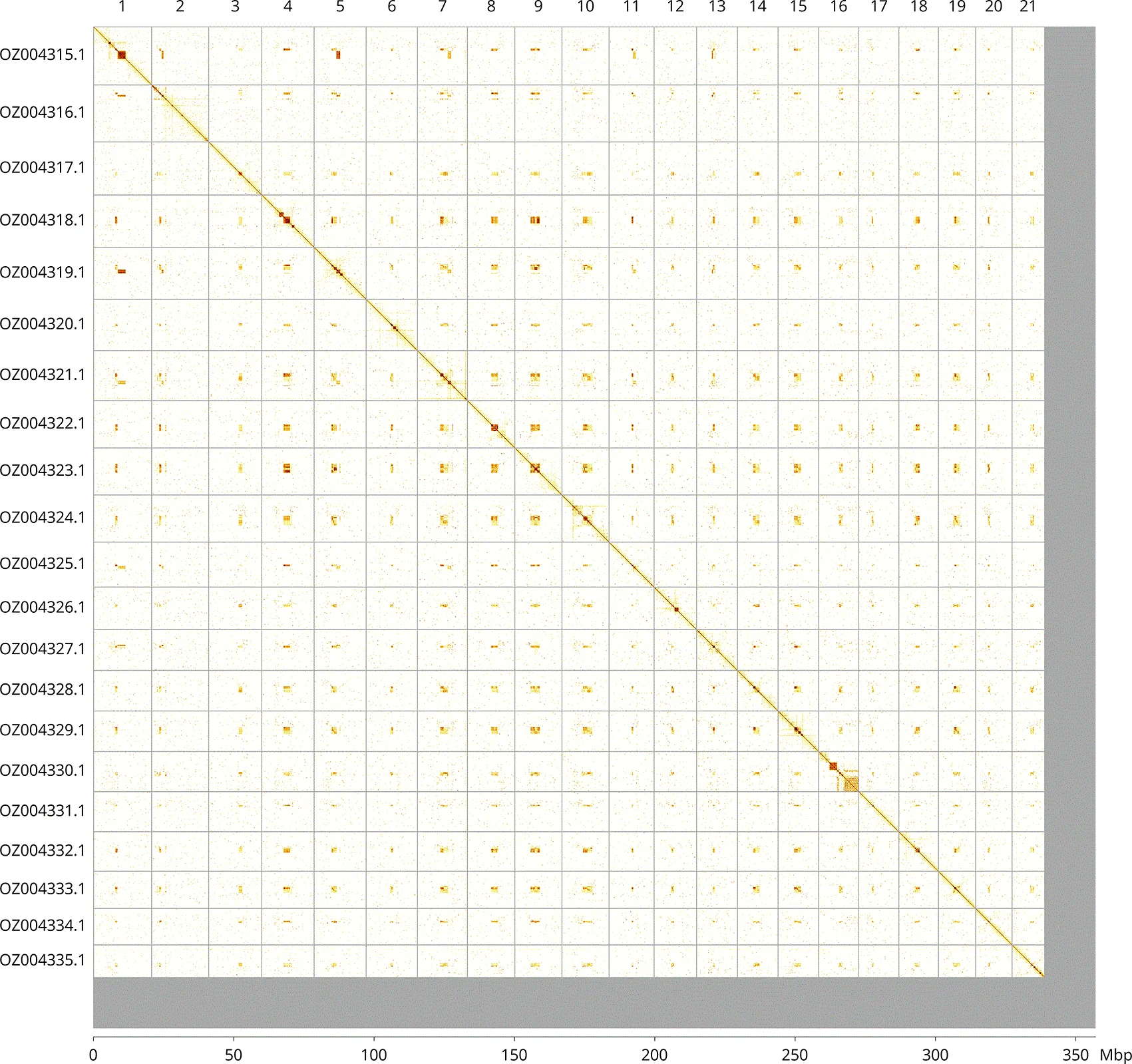
Hi-C contact map of the *Pelagia noctiluca* genome assembly. Assembled chromosomes are shown in order of size and labelled along the axes, with a megabase scale shown below. The plot was generated using PretextSnapshot.

Most of the assembly sequence (94.91%) was assigned to 21 chromosomal-level scaffolds. These chromosome-level scaffolds, confirmed by Hi-C data, are named according to size ( Figure 3; Table 3). During curation, we noted that the exact order and orientation of the centromeric repeat regions is unknown.

**Table 3.** Chromosomal pseudomolecules in the primary genome assembly of *Pelagia noctiluca* jsPelNoct2.

INSDC accession	Molecule	Length (Mb)	GC%
OZ004315.1	1	20.81	37.50
OZ004316.1	2	20.32	37.50
OZ004317.1	3	18.95	37
OZ004318.1	4	18.66	37
OZ004319.1	5	18.55	37
OZ004320.1	6	18.24	37
OZ004321.1	7	17.83	37.50
OZ004322.1	8	16.83	37
OZ004323.1	9	16.80	37
OZ004324.1	10	16.77	37.50
OZ004325.1	11	16.05	37
OZ004326.1	12	15.13	37.50
OZ004327.1	13	14.53	37
OZ004328.1	14	14.51	37.50
OZ004329.1	15	14.44	37
OZ004330.1	16	14.31	37
OZ004331.1	17	14.30	37
OZ004332.1	18	14.06	37
OZ004333.1	19	13.28	37
OZ004334.1	20	12.96	37
OZ004335.1	21	11.58	37

The mitochondrial genome was also assembled (length 16.7 kb, OZ004336.1). This sequence is included as a contig in the multifasta file of the genome submission and as a standalone record.

### Assembly quality metrics

**Figure 4.**
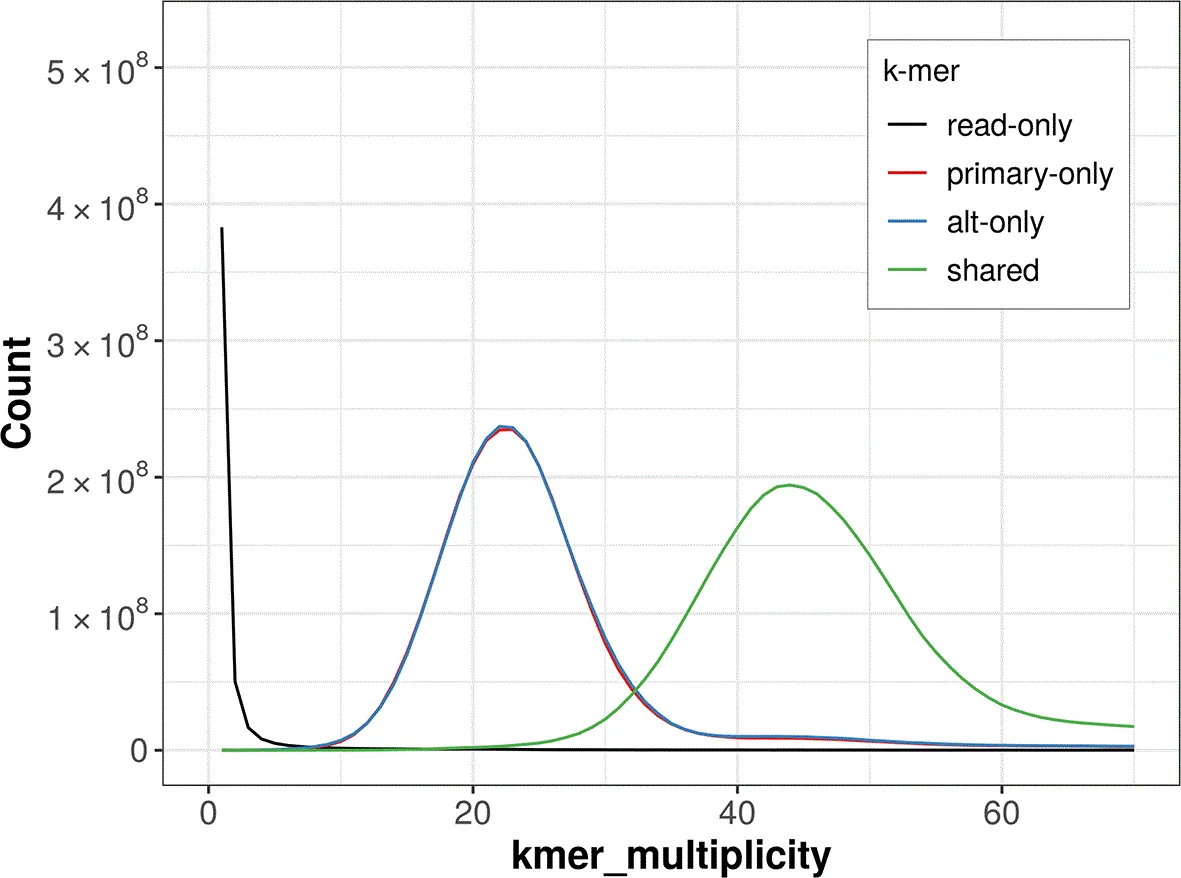
Evaluation of *k*-mer completeness using MerquryFK. This plot illustrates the recovery of *k*-mers from the original read data in the final assemblies. The horizontal axis represents *k*-mer multiplicity, and the vertical axis shows the number of *k*-mers. The black curve represents *k*-mers that appear in the reads but are not assembled. The green curve corresponds to *k*-mers shared by both assemblies, and the red and blue curves show *k*-mers found only in one assembly.

The primary assembly achieves an estimated QV of 61.2 and a *k*-mer completeness of 62.27%. The union of the primary assembly and alternate haplotig set achieves an estimated QV of 59.8 and a *k*-mer completeness of 99.54% ( Figure 4). The alternate haplotig set has an estimated QV of 59.4 and a *k*-mer completeness of 62.60%.

**Figure 5.**
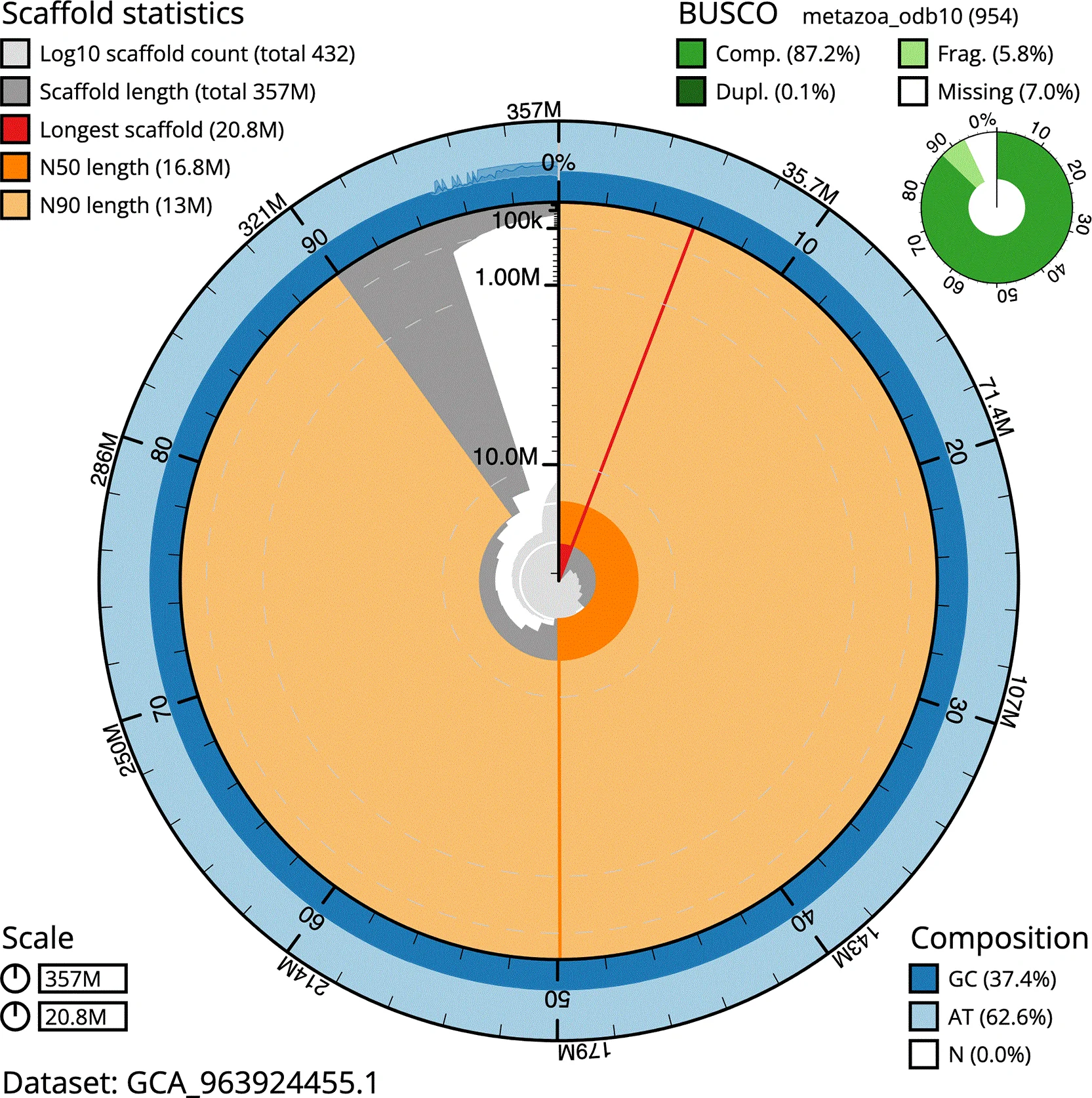
Assembly metrics for jsPelNoct2.1. The BlobToolKit snail plot provides an overview of assembly metrics and BUSCO gene completeness. The circumference represents the length of the whole genome sequence, and the main plot is divided into 1 000 bins around the circumference. The outermost blue tracks display the distribution of GC, AT, and N percentages across the bins. Scaffolds are arranged clockwise from longest to shortest and are depicted in dark grey. The longest scaffold is indicated by the red arc, and the deeper orange and pale orange arcs represent the N50 and N90 lengths. A light grey spiral at the centre shows the cumulative scaffold count on a logarithmic scale. A summary of complete, fragmented, duplicated, and missing BUSCO genes in the metazoa_odb10 set is presented at the top right. An interactive version of this figure can be accessed on the BlobToolKit viewer.

**Figure 6.**
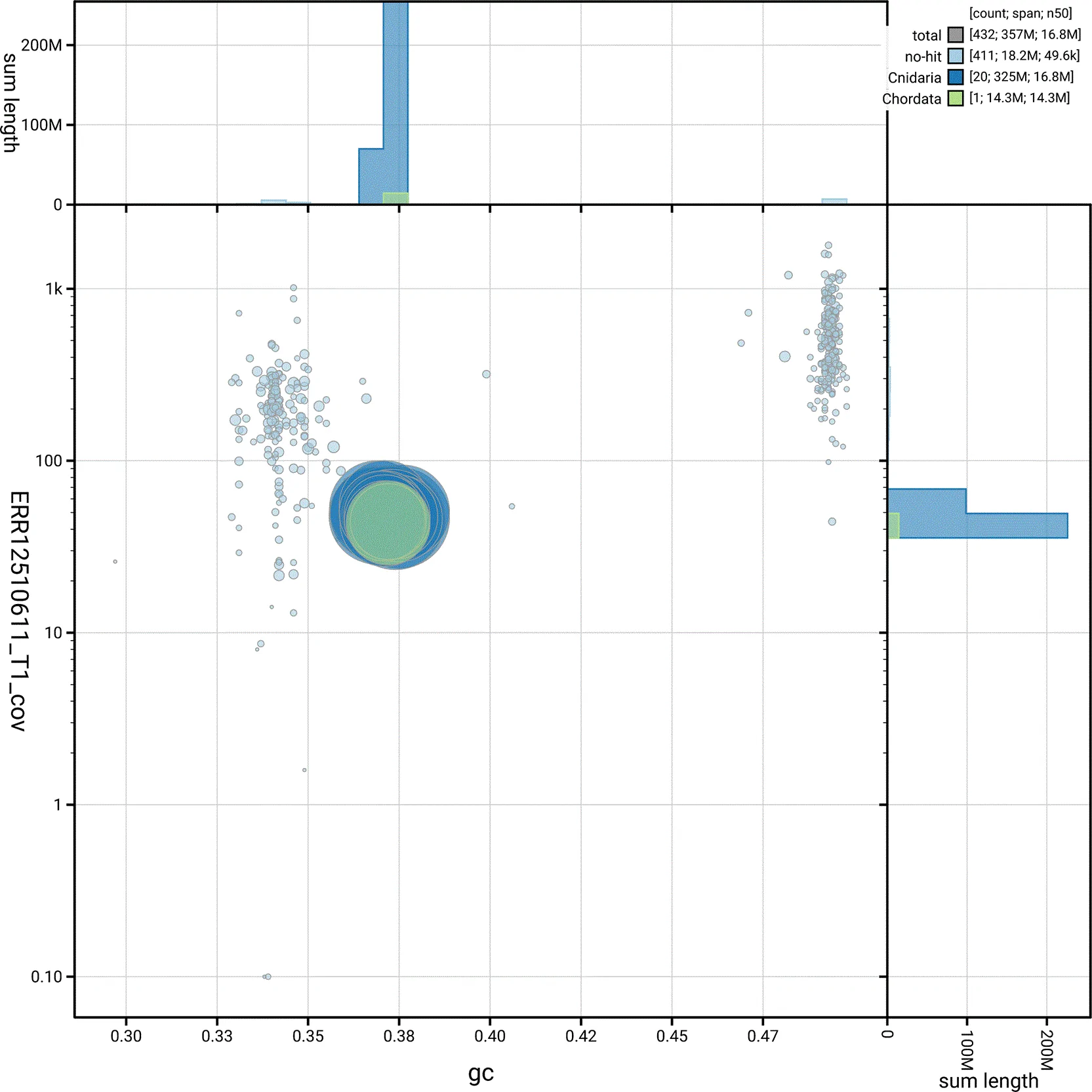
BlobToolKit blob plot for jsPelNoct2.1. The plot shows base coverage (vertical axis) and GC content (horizontal axis). The circles represent scaffolds, with the size proportional to scaffold length and the colour representing phylum membership. The histograms along the axes display the total length of sequences distributed across different levels of coverage and GC content. An interactive version of this figure is available on the BlobToolKit viewer.

BUSCO v.5.5.0 analysis using the metazoa_odb10 reference set ( *n* = 954) identified 87.2% of the expected gene set (single = 87.1%, duplicated = 0.1%). The snail plot in Figure 5 summarises the scaffold length distribution and other assembly statistics for the primary assembly. The blob plot in Figure 6 shows the distribution of scaffolds by GC proportion and coverage.

Table 4 lists the assembly metric benchmarks adapted from Rhie *et al.* (2021) and the Earth BioGenome Project Report on Assembly Standards January 2026. The EBP metric, calculated for primary assembly, is **6.C.Q61**, meeting the recommended 6.C.Q40 reference standard.

**Table 4.** Earth Biogenome Project summary metrics for the *Pelagia noctiluca* assembly.

Measure	Value	Benchmark
EBP summary (primary)	6.C.Q61	6.C.Q40
Contig N50 length	6.03 Mb	≥ 1 Mb
Scaffold N50 length	16.77 Mb	= chromosome N50
Consensus quality (QV)	Primary: 61.2; alternate haplotig set: 59.4; primary plus alternate haplotig set: 59.8	≥ 40
*k*-mer completeness	Primary: 62.27%; primary plus alternate haplotig set: 99.54%; alternate haplotig set: 62.60%	> 90%
BUSCO	C:87.2% [S:87.1%, D:0.1%], F:5.8%, M:7.0%, n:954	S > 90%; D < 5%
Percentage of assembly assigned to chromosomes	94.91%	≥ 90%

**Notes:** The EBP summary uses log10(Contig N50); chromosome-level (C) or log10(Scaffold N50); Q (Merqury QV). BUSCO: C=complete; S=single-copy; D=duplicated; F=fragmented; M=missing; n=orthologues.

**Table 5.** Software versions and sources used for *Pelagia noctiluca.*

Software	Version	Source
BLAST	2.14.0	https://ftp.ncbi.nlm.nih.gov/blast/executables/blast+/
BlobToolKit	4.3.3	https://github.com/blobtoolkit/blobtoolkit
BUSCO	5.5.0; 5.4.3	https://gitlab.com/ezlab/busco
bwa-mem2	2.2.1	https://github.com/bwa-mem2/bwa-mem2
DIAMOND	2.1.8	https://github.com/bbuchfink/diamond
fasta_windows	0.2.4	https://github.com/tolkit/fasta_windows
FastK	1.1	https://github.com/thegenemyers/FASTK
GenomeScope2.0	2.0.1	https://github.com/tbenavi1/genomescope2.0
Gfastats	1.3.6	https://github.com/vgl-hub/gfastats
Hifiasm	0.19.8-r603	https://github.com/chhylp123/hifiasm
HiGlass	1.13.4	https://github.com/higlass/higlass
MerquryFK	1.1.0-c1	https://github.com/thegenemyers/MERQURY.FK
Minimap2	2.24-r1122	https://github.com/lh3/minimap2
MitoHiFi	2	https://github.com/marcelauliano/MitoHiFi
MultiQC	1.14; 1.17 and 1.18	https://github.com/MultiQC/MultiQC
Nextflow	23.04.1; 23.10.0	https://github.com/nextflow-io/nextflow
PretextSnapshot	0.0.4	https://github.com/sanger-tol/PretextSnapshot
PretextView	1.0.3	https://github.com/sanger-tol/PretextView
purge_dups	1.2.3	https://github.com/dfguan/purge_dups
samtools	1.16.1; 1.17; 1.18	https://github.com/samtools/samtools
sanger-tol/ascc	0.1.0	https://github.com/sanger-tol/ascc
sanger-tol/blobtoolkit	0.3.0	https://github.com/sanger-tol/blobtoolkit
sanger-tol/curationpretext	1.4.2	https://github.com/sanger-tol/curationpretext
Seqtk	1.4-r122	https://github.com/lh3/seqtk
Singularity	3.9.0	https://github.com/sylabs/singularity
TreeVal	1.0.2	https://github.com/sanger-tol/treeval
YaHS	1.1a.2	https://github.com/c-zhou/yahs

### Genome annotation report

The *Pelagia noctiluca* genome assembly (GCA_963924455.1) was annotated by Ensembl at the European Bioinformatics Institute (EBI). This annotation includes 102 026 transcribed mRNAs from 19 573 protein-coding and 72 437 non-coding genes. The average transcript length is 2 802.89 bp, with an average of 1.11 coding transcripts per gene and 2.83 exons per transcript. Further details of this annotation are available from the Ensembl annotation page.

## Author information

Contributors are listed at the following links:

• Members of the Wellcome Sanger Institute Tree of Life Management, Samples and Laboratory team
• Members of Wellcome Sanger Institute Scientific Operations – Sequencing Operations
• Members of the Wellcome Sanger Institute Tree of Life Core Informatics team
• Members of the EBI Aquatic Symbiosis Genomics Data Portal Team
• The Aquatic Symbiosis Genomics Project leadership

## Wellcome sanger institute – legal and governance

The materials that have contributed to this genome note have been supplied by a Tree of Life collaborator. The Wellcome Sanger Institute employs a process whereby due diligence is carried out proportionate to the nature of the materials themselves, and the circumstances under which they have been/are to be collected and provided for use. The purpose of this is to address and mitigate any potential legal and/or ethical implications of receipt and use of the materials as part of the research project, and to ensure that in doing so we align with best practice wherever possible.

The overarching areas of consideration are:

• Ethical review of provenance and sourcing of the material
• Legality of collection, transfer and use (national and international)

Each transfer of samples is undertaken according to a Research Collaboration Agreement or Material Transfer Agreement entered into by the Tree of Life collaborator, Genome Research Limited (operating as the Wellcome Sanger Institute) and in some circumstances other Tree of Life collaborators.

## Data Availability

European Nucleotide Archive: Pelagia noctiluca. Accession number PRJEB71881; https://identifiers.org/ena.embl/PRJEB71881. The genome sequence is released openly for reuse. The *Pelagia noctiluca* genome sequencing initiative is part of the Aquatic Symbiosis Genomics Project (PRJEB43743) and the Sanger Institute Tree of Life Programme (PRJEB43745). All raw sequence data and the assembly have been deposited in INSDC databases. Raw data and assembly accession identifiers are reported in Tables 1 and 2. Production code used in genome assembly at the WSI Tree of Life is available at https://github.com/sanger-tol . Table 5 lists software versions used in this study.
