# Supplementary material for: The chromosomal genome sequence of the mauve stinger jellyfish, Pelagia noctiluca (Forsskål, 1775) (Semaeostomeae: Pelagiidae)
Source: Wellcome Open Res. 2026 Aug 27;11:552. [Version 1] doi: 10.12688/wellcomeopenres.27407.1 (PMC13615355; doi:10.12688/wellcomeopenres.27407.1)
Supplement: Peer review history

# Reviewer report: Reviewer response for version 1

**DOI:** 10.21956/wellcomeopenres.30167.r166183

**Published:** 2026-09-26

**Authors:** Adrián Jaimes-Becerra

**Affiliations:** 1 Tohoku University, Sendai, Japan

This paper presents a reference genome from a single adult female *Pelagia noctiluca*. The authors combined PacBio HiFi sequencing, Hi-C, and RNA-seq to produce a 357.08 Mb primary assembly, with 94.91% of the sequence assigned to 21 chromosome-scale scaffolds. This agrees with the chromosome number previously reported from Karyotypic analyses for the species. They also report a 16.7 kb mitochondrial genome and an Ensembl annotation containing 19,573 protein-coding genes. This is a valuable resource for scyphozoan research, and the laboratory and computational work is conducted carefully.

 I have two requests for clarification:

 -Complete BUSCO recovery in the primary assembly is 87.2%. Please discuss possible reasons for the missing BUSCO genes.

 -The manuscript reports 16.18 Gb of PacBio sequence data from 1.57 million reads used for assembly. Please clarify whether these values refer to the data before filtering or to the reads retained after filtering.

Are sufficient details of methods and materials provided to allow replication by others?

Yes

Is the rationale for creating the dataset(s) clearly described?

Yes

Are the datasets clearly presented in a useable and accessible format?

Yes

Are the protocols appropriate and is the work technically sound?

Yes

Reviewer Expertise:

Evolutionary biology, Cnidaria, Bioinformatics.

I confirm that I have read this submission and believe that I have an appropriate level of expertise to confirm that it is of an acceptable scientific standard.

# Reviewer report: Reviewer response for version 1

**DOI:** 10.21956/wellcomeopenres.30167.r166181

**Published:** 2026-09-25

**Authors:** Shawn M Burgess

**Affiliations:** 1 National Human Genome Research Institute, Bethesda, USA

This report describes the assembly of the mauve stinger genome. This jellyfish is broadly distributed in the earth's oceans and seas indicating it has highly adaptive properties. A full genome assembly is a foundation for many useful studies involving this animal. The methods are clear and appropriate for a quality assembly. The assembly statistics are good, indicating a high quality assembly. I have no reservations for this manuscript.

Are sufficient details of methods and materials provided to allow replication by others?

Yes

Is the rationale for creating the dataset(s) clearly described?

Yes

Are the datasets clearly presented in a useable and accessible format?

Yes

Are the protocols appropriate and is the work technically sound?

Yes

Reviewer Expertise:

Genomics, genetics, genome assembly, regeneration, zebrafish, model organism development.

I confirm that I have read this submission and believe that I have an appropriate level of expertise to confirm that it is of an acceptable scientific standard.

# Reviewer report: Reviewer response for version 1

**DOI:** 10.21956/wellcomeopenres.30167.r166177

**Published:** 2026-09-15

**Authors:** Kiyoshi Naruse

**Affiliations:** 1 Laboratory of Bioresources, National Institute for Basic Biology, Okazaki, Japan

**Reviewer comments**

 This manuscript presents the chromosome-level genome assembly and annotation of the mauve stinger jellyfish, *Pelagia noctiluca* (Forsskål, 1775; jsPelNoct2.1). The assembly appears robust: the HiFi contig N50 is 6.03 Mb, Hi-C scaffolding produced a scaffold N50 of 16.77 Mb, the major scaffolds correspond to the expected chromosome number, and 94.91% of the 357.08 Mb assembly is assigned to chromosome-scale pseudomolecules. The genome-wide Hi-C contact map provides convincing support for chromosome-scale organization, with no obvious large-scale misjoins or interchromosomal misassignments. Overall, the nuclear genome assembly can be considered a good chromosome-level resource.

 The main issue concerns the completeness and documentation of the gene annotation. BUSCO analysis using metazoa_odb10 recovered C:87.2% [S:87.1%, D:0.1%], F:5.8%, and M:7.0% (n = 954). The extremely low duplicated BUSCO proportion is reassuring and indicates that the gene set is not inflated by redundant haplotype-derived models. However, the complete score is below the commonly targeted 90% level, and the 12.8% fragmented or missing BUSCOs suggest that the annotation remains improvable.

**Major comments**

**1. Distinguish assembly and annotation completeness.**

 The authors should state clearly whether the reported BUSCO result was obtained in protein, transcriptome, or genome mode. BUSCO should also be performed in genome mode on the final nuclear assembly using the same metazoa_odb10 dataset. Comparing genome-mode and protein-mode BUSCO results will determine whether the fragmented/missing BUSCOs reflect limitations of the assembly or incomplete gene models.

**2. Assess fragmented and missing BUSCO loci.**

 The authors should investigate the approximately 55 fragmented and 67 missing BUSCOs. A supplementary table should report their genome-mode status, chromosome/scaffold location, proximity to gaps or contig boundaries, local repeat content, HiFi read depth, RNA-seq support, and protein-homology support. This would clarify whether the incomplete BUSCO score arises from gene-prediction failure, conservative filtering, repeat-associated assembly difficulty, or genuine divergence/loss.

**3. Report annotation evidence provenance.**

 Because the Ensembl Gene Annotation Pipeline integrates multiple evidence sources, it is not necessary to generate separate homology-only and RNA-seq-only annotations. However, the manuscript should report the proportion of final gene models supported by: same-species RNA-seq, RNA-seq splice junctions, protein or transcript homology, both RNA-seq and homology, and neither evidence type. This information is important for interpreting the moderate BUSCO completeness.

**4. Describe RNA-seq evidence.**

 The authors should provide details of the RNA-seq data used for annotation, including tissue and developmental/life-history stage, specimen number, sequencing depth, library type, mapping statistics, and the proportion of final models supported by transcript evidence. A limited range of tissues or stages may explain why some conserved genes are fragmented or absent from the final annotation.

**5. Provide independent HiFi coverage QC.**

 A chromosome-wide HiFi read-depth plot would strengthen the assembly assessment. The authors should show window-based median coverage across all pseudomolecules, with chromosome boundaries and approximately 0.5× and 2× coverage reference lines. This would help identify residual haplotigs, collapsed repeats, local anomalies, or high-coverage organellar sequences. The mapping and filtering strategy should be stated.

**6. Characterize residual sequences.**

 The remaining 5.09% of unplaced sequence should be summarized by scaffold length, GC content, HiFi coverage, repeat content, gene content, and taxonomic assignment. Putative mitochondrial sequences should be identified and reported separately from nuclear assembly statistics. A GC–coverage–taxonomy analysis would also help exclude bacterial, symbiont, prey-derived, or other contaminating scaffolds.

**Overall assessment**

 The HiFi/Hi-C assembly of *P. noctiluca* is structurally convincing and meets the criteria for a chromosome-level nuclear genome. The requested revisions should focus primarily on clarifying and improving the annotation-quality assessment, especially by comparing genome- and protein-mode BUSCO results, examining fragmented/missing BUSCO loci, and documenting the RNA-seq and homology evidence underlying the Ensembl gene models.

Are sufficient details of methods and materials provided to allow replication by others?

Yes

Is the rationale for creating the dataset(s) clearly described?

Yes

Are the datasets clearly presented in a useable and accessible format?

Yes

Are the protocols appropriate and is the work technically sound?

Yes

Reviewer Expertise:

Genetics and Genomics of fishes. Fish phylogeny and evolution of Oryzias species.

I confirm that I have read this submission and believe that I have an appropriate level of expertise to confirm that it is of an acceptable scientific standard, however I have significant reservations, as outlined above.

# Reviewer report: Reviewer response for version 1

**DOI:** 10.21956/wellcomeopenres.30167.r166178

**Published:** 2026-09-14

**Authors:** Yichen Dai

**Affiliations:** 1 Fudan University, Shanghai, China

The authors present a chromosome-level genome assembly for the mauve stinger jellyfish, *Pelagia noctiluca* , based on PacBio HiFi and Hi-C data, with RNA-seq used for annotation. This is a valuable data note for a non-model, ecologically important cnidarian. However, the assembly completeness and contamination evidence need stronger clarification before the genome can be considered fully validated.

 Major concerns:

 1. BUSCO completeness:

 The final BUSCO score is only 87.2% complete using metazoa_odb10. For a chromosome-level assembly, this is lower than expected. The genome size estimate from GenomeScope2 (358.68 Mb) is very close to the final assembly size (357.08 Mb), which makes the missing BUSCOs even more surprising.

 I suggest the authors look into this issue:

 (1) Can they report BUSCO scores after each major assembly step to validate which step might be the reason for this relatively low BUSCO score?

 (2) Can they test whether missing genes might be present in the alternate haplotig set, in unassembled reads, or in the no-hit scaffolds?

 (3) Do they think the missing genes are likely biological (e.g., lineage-specific losses), technical (assembly fragmentation), or caused by purging/curation?

 2. Manual curation:

 The manuscript states that manual curation included 28 breaks, 43 joins, and removal of 69 haplotypic duplications, reducing total assembly length by 10.1%. This is a substantial amount of sequence. To address this concern, the authors should provide the size and composition of the original primary assembly before cleaning.

 3. Decontamination:

 ASCC decontamination was performed after Hi-C scaffolding. If contaminants are scaffolded into chromosomes, they may be difficult to remove. More importantly, Figure 6 appears to show a Chordata-assigned sequence of approximately 14.3 Mb in the final assembly. If this is correct, it is a major concern: a 14.3 Mb chordate contaminant would be a relatively large fraction of the genome and could affect scaffolding and annotation.

 Can the authors:

 (1) Confirm whether the Chordata sequence is still present in the final assembly or whether the figure is from an earlier stage?

 (2) If this sequence was retained, why? Or has it been removed?

 (3) Report the pre- and post-ASCC assembly sizes and the taxa/proportions removed.

 (4) Clarify what the 18.2 Mb of “no-hit” sequence in Figure 6 is?

 4. *k* -mer completeness:

 Table 4 reports primary *k* -mer completeness of 62.27%, while the benchmark is >90%. The union of primary + alternate haplotigs reaches 99.54%, but the primary assembly alone does not. Similarly, BUSCO completeness is 87.2%, below the >90% benchmark. The claim that the assembly meets the “6.C.Q40 reference standard” is only true for the QV/contiguity component; it does not mean the assembly satisfies all EBP metrics. Which assembly (primary, alternate, or union) is being evaluated for each metric? How does the primary assembly reach 6.C.Q61?

 5. Non-coding genes:

 The Ensembl annotation reports 19,573 protein-coding genes and 72,437 non-coding genes. The number of non-coding genes is very high, is it possible that this is affected by repeats or fragmented transcripts? The authors should provide annotation quality metrics, such as BUSCO scores for the annotated protein set. Since they also have RNA-seq data, can they show the proportion of genes supported by RNA-seq evidence? In addition, will it be possible to show the number of transcripts with complete ORFs? What might be causing this very high non-coding gene number?

 Minor concern: Table 5 lists multiple versions for some of the software used (e.g. BUSCO). Where different versions used in different steps?

Are sufficient details of methods and materials provided to allow replication by others?

Yes

Is the rationale for creating the dataset(s) clearly described?

Yes

Are the datasets clearly presented in a useable and accessible format?

Yes

Are the protocols appropriate and is the work technically sound?

Yes

Reviewer Expertise:

Genome assembly, cross-species comparison

I confirm that I have read this submission and believe that I have an appropriate level of expertise to confirm that it is of an acceptable scientific standard, however I have significant reservations, as outlined above.

# Reviewer report: Reviewer response for version 1

**DOI:** 10.21956/wellcomeopenres.30167.r166176

**Published:** 2026-08-31

**Authors:** Abdelmalek Lekired

**Affiliations:** 1 University of New Mexico, Albuquerque, New Mexico, USA

**The chromosomal genome sequence of the mauve stinger jellyfish,** *Pelagia noctiluca* **(Forsskål, 1775) (Semaeostomeae: Pelagiidae)**

 The authors present a genome assembly from a specimen of *Pelagia noctiluca* (Mauve Stinger; Cnidaria; Scyphozoa; Semaeostomeae; Pelagiidae). The genome assembly has a total length of 357.08 Mb, with 94.91% of the assembly scaffolded into 21 chromosomal pseudomolecules. The mitochondrial genome was also assembled, with a length of 16.7 kb. Gene annotation performed by Ensembl identified 19,573 protein-coding genes.

**Report:**

 The authors present a chromosome-level genome assembly of the mauve stinger jellyfish, *Pelagia noctiluca* (Forsskål, 1775) (Semaeostomeae: Pelagiidae). The genome was sequenced using PacBio HiFi technology and scaffolded using Hi-C data generated with the Arima Genomics system.

 The results show that 94.91% of the final assembly was anchored into 21 chromosomal pseudomolecules.

 However, the final BUSCO analysis showed only 87% completeness based on the *metazoa_odb10* dataset. This relatively low level of completeness raises concerns regarding the assembly process and, particularly, the effects of the purging and decontamination steps. I recommend that the authors provide additional information and analyses to clarify the following points.

**1-** The GenomeScope2 analysis estimates a genome size of approximately 357.999 Mb, with an estimated heterozygosity of 3.01%. The final assembly, after cleaning, has a size of 357.08 Mb, which is remarkably close to the GenomeScope2 estimate. However, the final BUSCO analysis reports only 87% completeness. Given the close agreement between the estimated genome size and the final assembly size, the relatively low BUSCO completeness is unexpected. How do the authors explain this discrepancy? It would be useful to clarify whether the missing BUSCOs are attributable to genuine biological characteristics of the species, sequencing limitations, assembly fragmentation, or losses introduced during the cleaning/decontamination and haplotig-purging steps.

**2-** The authors indicate that cleaning and contaminant removal were performed after the Hi-C scaffolding step. Since 94.91% of the final assembly is anchored into 21 chromosomal pseudomolecules, it is important to clarify the origin and proportion of the sequences removed during decontamination. In particular, the manuscript should specify whether the reported approximately 10% of contaminant sequences removed was calculated relative to the final 357 Mb assembly or relative to the original primary assembly. The size and characteristics of the original assembly should also be reported so that the extent of sequence loss during the cleaning and decontamination steps can be evaluated.

**3-** The authors used Purge_Dups to remove duplicated sequences. However, Hifiasm already performs internal procedures for identifying and removing redundant/duplicated sequences during assembly. I therefore wonder whether the same duplicated haplotigs may have been removed at multiple stages of the workflow. The authors should clarify the exact sequence of duplication-removal steps, including which duplicated sequences were removed by Hifiasm and which were subsequently removed by Purge_Dups. It would also be useful to report the assembly size and BUSCO completeness after each major purging step to assess whether genuine genomic sequences may have been inadvertently removed.

**4-** I recommend that the authors provide BUSCO results for the primary Hifiasm assembly before haplotig purging, after Purge_Dups, and after Hi-C scaffolding and final cleaning/decontamination. This would allow readers to evaluate how each step affected genome completeness and determine whether the reduction in BUSCO completeness occurred during assembly, haplotig purging, scaffolding, or decontamination. I also recommend performing decontamination before Hi-C scaffolding, or, at minimum, providing a clear justification for performing decontamination after scaffolding. Removing contaminant sequences before scaffolding may help prevent non-target sequences from being incorporated into chromosomal pseudomolecules and would provide a cleaner starting point for the Hi-C scaffolding process.

Are sufficient details of methods and materials provided to allow replication by others?

Partly

Is the rationale for creating the dataset(s) clearly described?

Yes

Are the datasets clearly presented in a useable and accessible format?

Yes

Are the protocols appropriate and is the work technically sound?

Yes

Reviewer Expertise:

Invertebrate Genomics, RNAseq, Fungal Genomics, Bacterial Genomics, Assembly and annotation of non model organisms

I confirm that I have read this submission and believe that I have an appropriate level of expertise to confirm that it is of an acceptable scientific standard, however I have significant reservations, as outlined above.
